# Optional subject indexing in spontaneous speech in Modern Persian

**DOI:** 10.1515/ling-2023-0235

**Published:** 2026-02-20

**Authors:** Pegah Faghiri

**Affiliations:** Université Paris-Saclay, CNRS, Laboratoire Interdisciplinaire des Sciences du Numérique, 91400, Orsay, France

**Keywords:** person indexing, differential argument marking, spoken versus written modality, corpus linguistics, usage-based methods, Persian

## Abstract

This article presents a case of (non-canonical) optional subject marking observed exclusively in spontaneous speech. Persian is a pro-drop language with obligatory subject agreement marked by verbal suffixes and a set of clitic pronouns serving different functions, e.g., pronominal objects encliticized to the verb. In standard Persian, subject agreement for the 3rd person singular (3SG) is consistently realized by zero in past tenses; however, as shown in this study, in spoken language, speakers occasionally use the 3SG pronominal clitic =*eš* instead of zero. This non-standard usage is generally neglected, if not ignored, in previous research. Drawing on evidence from historical studies and naturally produced data from spoken and transcribed Persian, this study highlights the empirical shortcomings of previous claims that the subject agreement marker -*eš* results from grammaticalization of the object clitic =*eš* as a means of repairing or levelling the verbal agreement paradigm. Instead, it is argued that while written Persian preserves more parsimonious constructions, the redundant use of -*eš* to index the subject, similar to object clitic doubling, is part of the indexing system of spoken Persian and can be understood in terms of fluid differential subject marking. Differences in indexing between the written (i.e., offline language production) and spoken (i.e., spontaneous language production) modalities are not unusual and may be caused by different factors. Studying these differences in transcribed spoken data, this study identifies distinct patterns of information packaging in the two modalities and offers an explanation in terms of cognitive principles of language processing and production to account for the difference in indexing between spoken and written Persian.

## 1 Introduction

Persian is a pro-drop language with obligatory subject agreement realized by verbal suffixes. In standard Persian, subject agreement for the 3rd person singular is realized by a suffix in present tenses, while it is unmarked in past tenses. In colloquial Persian, however, speakers sometimes use the 3rd person singular pronominal enclitic *=eš* instead of zero. This use is not endorsed by prescriptive grammars and described as a “new trend” by one of the few existing studies (Rasekh 2014: 25). It could indeed be stigmatized, and even linguistic researchers are inclined to ignore it: in a richly annotated spoken corpus (Adibifar 2016) this subject marker is clearly heard but it does not get transcribed. Descriptive reference grammars do, however, mention this use as a particularity of colloquial or spoken Persian (e.g., Lazard 1957; Paul 2019; Vahidian Kamyar 1964, 2005). The present study is a first usage-based investigation of this non-standard optional subject marker, on the variety spoken in Iran, also known as *Farsi*.1Modern Persian, written with Arabic script, is established in the Iranian territory as a language of literature, science and administration since the 11th century. It emerged after the Arabic conquest in the 7th century, ending the Sassanid dynasty and its language of administration, Middle Persian. Modern Persian has a number of varieties (e.g., Dari spoken in Afghanistan and Tajik spoken in Tajikistan). In Iran, it is the only official language and the only language of the educational system. According to Gilbert Lazard, standard Modern Persian dates back to the 13th century (“[la langue] que décrivait la plupart des grammaires et qui s’offre, à peu près fixée, dans la prose littéraire postérieur au XIIIème siècle et antérieure aux temps modernes” (Lazard 1963: 24)). For details, see, e.g., Paul 2019 (569–580).


The few theoretical studies that have discussed this marker so far focus on its clitichood (clitic vs. affix/agreement marker), aiming to contribute to the ongoing debate on pronominal clitics in Persian and more generally across languages (Jügel and Samvelian 2016; Mahootian and Gebhardt 2018; Rasekh 2014). In this study, I treat subject-marking -*eš* as an affix; however, while I will present some empirical data to justify my choice, I do not engage with this debate. In other words, my claims and findings do not bear on my view of the affix- or clitichood of -*eš*.

Up to now, *-eš* has been viewed as a subject agreement marker resulting from the grammaticalization of the object clitic =*eš*, as a means of ‘repairing’ or ‘harmonizing’ the verbal agreement paradigm. I show that this account is not empirically justified. On the contrary, all empirical evidence suggests that the optional use of -*eš* as a sub-standard construction has been part of the indexing system in spoken Persian since its early stages,2I use the terms “index” and “indexing” in line with Haspelmath (2013) – who proposed the term “indexes” following Lazard (1998) to refer to “bound person forms”, encompassing affixes and clitics. Accordingly, any core argument, i.e., the subject and the object, may be indexed on the verb by affixes or clitics. Hence subject agreement mentioned earlier is a subtype of indexing. In other words, the indexing system in Persian includes both subject agreement affixes and object enclitics. In this paper, the term *agreement* is used when applicable, i.e., the systematic indexing of the subject on the verb via personal suffixes, and *indexing* otherwise. while – as all previous studies agree – the written register consistently excludes this use of -*eš*. I consider this to be a crucial point for the issue at stake. Indeed, it is well-established that spoken and written modalities differ in many respects, in particular in the organization of discourse. What is referred to more specifically by spoken and written modalities here is the difference between spontaneous *real time impromptu* language production and *offline* language production, where there is time for choosing between alternative constructions and expressions as well as the possibility for rephrasing (Miller and Fernandez-Vest 2006). Efficient communication does not manifest itself in the same way in these two modalities of language production. Where the written modality prefers (and affords) parsimonious and simple constructions, redundancy is common in spontaneous speech (see, e.g., Degen et al. 2020 and references therein). In a methodologically new approach, I use data from transcribed spoken Persian to uncover patterns of difference between these two modalities. This particular type of data allows for comparing utterances produced spontaneously in real time with their written equivalents. The latter are well-considered sentences produced offline and at a different time by transcribers. These patterns, I argue, show that written Persian prefers parsimonious constructions, whereas in spoken Persian, redundant indexing is common.3The short labels “written” and “spoken” language are used throughout the text for convenience. Furthermore, given their relevance in the context of Persian, the labels “standard” and “colloquial” registers are also used as shortcuts for “written standard” and “spoken colloquial”. It is indeed possible to use standard Persian in the spoken modality or colloquial Persian in the written modality. These are, however, beyond the scope of the present paper.


I further show that the use of subject-marking -*eš* is not a new trend in Modern Persian, and that the historical evidence actually points to the opposite: the evolution of the agreement system in West-Iranian languages (see, e.g., Haig 2017, 2018; Jügel and Samvelian 2016; Lazard 1963) suggests that this use of -*eš* for indexing the 3rd person singular subject may be a relic of an earlier stage of the agreement system. However, historical and/or regional variation can only explain the existence of zero/-*eš* alternation, not how it works synchronically. In short, this alternation has not been systematically studied, and, consequently, its triggers remain obscure.

While it is possible to delimit the configuration and, to some extent, the speech context in which -*eš* can be used to index the 3rd person singular subject, no (inherent or non-inherent) factor is known to require it, i.e., there is no context in which dropping -*eš* (i.e., zero marking) could be considered ungrammatical or unacceptable. Yet, a basic examination of available spoken corpora easily shows that -*eš* is far from sporadic in colloquial Persian.

Framing the zero/-*eš* alternation in terms of Differential Argument Indexing (DAI) – that is, as a case of Differential Argument Marking (DAM, e.g., Seržant and Witzlack-Makarevich 2018), or Differential Subject Marking (DSM, e.g., de Hoop and de Swart 2008) –  it is argued that only a systematic study of subject realization in discourse can shed light on its triggers. These may include lexico-semantic properties of the verb, as well as discourse-related factors, and may involve a degree of sociolinguistic variation. There may be no (more or less) straightforward rule or set of strict constraints or delineated contexts, as for example is the case for Persian’s well-known Differential Object Marking (DOM). Indeed, it is clear that the zero/-*eš* alternation is not triggered by grammatical rules or ‘hard’ constraints. Nevertheless, there may be a set of ‘soft’ constraints or (statistical) preferences or contexts (dis)favouring the presence of -*eš*.

The remainder of the article is structured as follows: a brief overview of Differential Argument Indexing is provided in Section 2.1, and an outline of the agreement system in spoken and written Persian with a focus on -*eš* is given in Section 2.2. I discuss the nature of subject-marking -*eš* in terms of affix-/clitichood in Section 2.3, and summarize existing claims on the use of -*eš* as a subject marker in Section 2.4; the research questions are presented in Section 3; the data and methods are described in Section 4; in light of usage data, I underline previous descriptive shortcomings in Section 5; in Section 6, I highlight the importance of the spoken modality in the issue at stake. Section 7 provides a theoretical discussion justifying the analysis proposed in this study against previous accounts.

## 2 Background

### 2.1 Differential Argument Indexing

In this study the optional indexing of the 3rd person subject in Persian is framed in terms of Differential Argument Indexing (e.g., Walker et al. 2024), a label which I find particularly appropriate since it covers both subjects and objects.4In typology, more cross-linguistically relevant labels, S, A and P, are used to refer to verbal arguments. Modern Persian has nominative-accusative alignment, for which the labels subject and object are broadly used in the literature. This paper follows the latter for convenience and only uses S/A/P where relevant. DAI is used here as a subtype of DAM, which encompasses differential marking of core arguments both via their indexing on the verb (head-marking) and their flagging (dependent-marking). DAM can be either “symmetrical”, i.e., showing an alternation between two different markers, or “asymmetrical”, i.e., showing an alternation between an overt marker and zero. DAM alternations may be conditioned by a wide range of factors, including inherent (e.g., person, animacy) and non-inherent (e.g., definiteness, topicality) properties of arguments, event semantics (e.g., volitionality, affectedness), verb classes, and other properties of the clause such as TAM or polarity (Witzlack-Makarevich and Seržant 2018, and references therein; also see Walker 2024 for a more recent overview).

DAM may be “split”, i.e., have absolute conditioning factors, or be “fluid”, i.e., a matter of (statistical) preferences or both (Witzlack-Makarevich and Seržant 2018: 27–29). In split DAM, marking options are in strict complementary distribution, whereas in fluid DAM they are not. Persian’s DOM is an interesting combining case, labelled as “split-fluid” by Witzlack-Makarevich and Seržant (2018: 28). It is ruled by a necessary but not sufficient condition based on definiteness: definite (direct) objects are necessarily marked (by the enclitic *=rā*), whereas non-definite objects are not always unmarked. Some contexts require the obligatory marking of non-definite objects, but there are other contexts in which *rā*-marking is more fluid, with a set of factors favouring the presence of *=rā* (see, e.g., Lazard 1957).

Persian displays another interesting case of split-fluid DAM, which concerns the indexing of the 3rd person plural subject and occurs in both colloquial and formal registers. As illustrated in (1), indefinite (inanimate) 3rd person plural subjects can trigger both singular or plural agreement on the verb. Or, as Haig (2018: 789) put it, these subjects “*optionally* fail to trigger the expected plural agreement on the verb” [emphasis is mine].

(1)

**Table:** 

*čerāq-hā*	*xāmuš*	*mi-šav-ad/and*
light-pl	off	ipfv-become.prs-3sg/3pl
‘Lights will be turned off.’		

Similar to the zero/-*eš* alternation in person indexing, and unlike Persian’s DOM, it is hard, if not impossible, to predict the choice between singular/plural agreement for a given utterance. Nevertheless, it is possible to delimit which plural subjects allow for the alternation, and to identify the factors that favour the absence of plural agreement.

### 2.2 Agreement in Persian and the uses of *eš*

Persian is a pro-drop language with obligatory subject agreement realized by verbal suffixes (see Table 1). In standard Persian, subject agreement for 3SG is marked by the suffix -*ad* in present (2a) and by zero in past tenses (2b). There is also a set of enclitic pronouns, including the 3SG =*aš*, and pronominal objects can be cliticized to the verb (3).5Note that object is used here in its broad sense and includes direct and indirect/oblique objects. Moreover, the use of enclitic pronouns is not limited to verbal arguments, they can also be possessive pronouns, e.g., *ketāb=eš* ‘his/her book’, instead of full pronouns used in the Ezafe construction, *ketāb=e u* ‘his/her book’. This paradigm is also used for subject agreement, in both past and present tense, in the so-called experiencer constructions (see Jügel and Samvelian 2020), which display a form of non-canonical subject marking (4).6Non-canonical because (i) the agreement marker is not attached to the verb, (ii) the verb is in 3rd person singular regardless of person/number of the experiencer (viewed as the subject/S-argument). Another analysis would be to take the other nominal element, i.e., *xāb* in (3), as the (grammatical) subject of the verb.


**Table 1:** Person indexing in Standard and Colloquial Persian.

	Verb endings/suffixes				Clitics	
	Present stems		Past stems			
	Standard	Colloquial	Standard	Colloquial	Standard	Colloquial
SG						
1	-am	-am	-am	-am	=am	=am
2	-i	-i	-i	-i	=at	=et
3	-ad	-e	Ø	Ø/-eš	=aš	=eš
PL						
1	-im	-im	-im	-im	=emān	=emun
2	-id	-in	-id	-in	=etān	=etun
3	-and	-an	-and	-an	=ešān	=ešun

(2)

a.

**Table:** 

*Ali*	*man=rā*	*mi-bin-ad*
Ali	1sg=dom	ipfv-see.prs-3sg
‘Ali sees me.’

b.

**Table:** 

*Ali*	*man=rā*	*did*
Ali	1sg=dom	see.pst.3sg
‘Ali saw me.’

(3)

**Table:** 

*Ali*	*mi-bin-ad=aš*
Ali	ipfv-see.prs-3sg=3sg
‘Ali sees him/her/it.’

(4)

a.

**Table:** 

*Ali*	*xāb=aš*	*bord*
Ali	sleep=3sg	take.pst.3sg
‘Ali fell asleep (was taken by sleep).’

b.

**Table:** 

*man*	*dār-ad*	*xāb=am*	*mi-bar-ad*
1sg	aux.prs-3sg	sleep=1sg	ipfv-take.prs-3sg
‘I am falling asleep.’

In colloquial speech, the paradigm is essentially the same, but with some phonological differences, e.g., *aš* versus *eš*. Importantly, speakers may use -*eš* to index the subject with past stems (5).7It is important to note that =*eš* has other (non-verbal) uses in colloquial speech. Namely, it can be used as a form of definite marker (i), seemingly as an extension of the possessive meaning (see footnote 5). To my knowledge this use has not yet been systematically described in the literature.

(i)

**Table:** 

*film=eš*	*xub*	*na-bud*
movie=3sg	good	neg-cop.pst.3sg
‘The movie was not good.’

Note that word order in sentences (5a) and (5b) indicate that they are from the colloquial register, whereas standard Persian requires verb-final order, as in (5c).

(5)

a.

**Table:** 

*Ali*	*raft-eš*	*birun*
Ali	go.pst.3sg-3sg	out

b.

**Table:** 

*Ali*	*raft*	*birun*
Ali	go.pst.3sg-3sg	out

c.

**Table:** 

*Ali*	*(be) birun*	*raft*
Ali	to out	go.pst.3sg-3sg
‘Ali went out.’

While (5a) is a typical colloquial example illustrating this sub-standard use of -*eš* (see, e.g., Rasekh 2014), an intransitive predicate with a human subject is not a necessary condition for this use, as shown in (6). Indeed, -*eš* occurs in a variety of configurations as the examples provided in this study aim to show (see Section 5).

(6)

a.

**Table:** 

*tu*	*kafš*=*am*	*sang*	*raft-eš*
in	shoe=1sg	stone	go.pst.3sg-3sg
‘There is a stone in my shoe (lit. stone is gone into my shoe).’

b.

**Table:** 

*xat*	*umad-eš*
line/tails	come.pst.3sg
‘(Result of coin-flipping) is/we have tails (lit. line came).’

c.

**Table:** 

*bārun (…) mi-umad*		*amā (…) qat*	*šod-eš*
rain	ipfv.come.pst.3sg	but	stop become.pst.3sg-3sg
‘It was raining (…) but (…) it stopped (raining).’			
			(Tehran English-Persian parallel corpus)

### 2.3 Morphosyntactic properties: affix or clitic?

There are no phonological differences between the pronominal enclitic =*eš* and its use as subject maker, and discussing the clitic-/affixhood of the latter is beyond the scope of this study. Nevertheless, I build on some morphosyntactic differences between the two uses of *eš,* treating subject-marking *eš* as an affix and the object marker *eš* as a clitic.

Clitic pronouns display a degree of flexibility/mobility with respect to the host that subject-marking -*eš* does not allow. This is evident, for instance, in the case of multiword verbal expressions, e.g., *daɁvat kardan* ‘invite’ (invitation do). In these cases, as illustrated in (7), *eš* can attach both to the (light) verb (7a), and to the noun (7b). Yet, if it is attached to the noun, it can only correspond to the object. Hence, while (7a) is ambiguous, (7b) is not. Note that attaching =*eš* to the noun is not possible with a non-referential object, as illustrated by (7c).

(7)

a.

**Table:** 

*Nima*_*i*_	*Ali*_*j*_ *=ro*	*daɁvat*	*kard-/=eš*_*i/j*_
Nima	Ali=dom	invitation	do.3sg-/=3sg

b.

**Table:** 

*Nima*_*i*_	*Ali*_*j*_ *=ro*	*daɁvat=eš*_j/*i_	*kard*
Nima	Ali=dom	invitation=3sg	do.pst.3sg
‘Nima invited Ali.’

c.

**Table:** 

*Nima_*i*_	*mehmun*_*j*_	*daɁvat=eš*_*i*_	*kard*
Nima	guest	invitation=3sg	do.pst.3sg
(intended) ‘Nima invited some guests.’

Another way to illustrate this difference is by comparing the active voice *daɁvat kardan* ‘invite (invitation do)’ with the passive *daɁvat šodan* ‘be invited (invitation become)’, as in (8). Only in the active voice can *eš* attach to both the noun and the verb.

(8)

a.

**Table:** 

*Nima*_*i*_	*Ali*_*j*_ *=ro*	*daɁvat=eš*_*j*_	*kard*
Nima	Ali=dom	invitation=3sg	do.pst.3sg
‘Nima invited Ali.’

b.

**Table:** 

*Ali	*be mehmuni*	*daɁvat=eš*	*šod*
Ali	to party	invitation=3sg	become.pst.3sg
(intended) ‘Ali was invited to the party.’

c.

**Table:** 

*Ali*	*be mehmuni*	*daɁvat*	*šod-eš*
Ali	to party	invitation	become.pst.3sg-3sg
‘Ali was invited to the party.’

These examples show that when *eš* is used as a subject marker, its placement is more restricted than when it is used as an object marker. Limited mobility is a hallmark of affixhood, and this is the only formal distinction one can establish between the two functions of *eš* at this stage. Hence, in order to avoid confusion, I refer to the subject marker *eš* using an affixal notation, while the object marker is treated as a clitic, in line with the existing literature.

### 2.4 Previous claims on subject-marking -*eš*

In the existing literature three publications include subject-marking -*eš* in their study of pronominal clitics in Persian: Jügel and Samvelian (2016), Mahootian and Gebhardt (2018), and Rasekh (2014). Jügel and Samvelian (2016) study the distribution of pronominal clitics in West-Iranian languages, including Persian, focusing on their different developmental paths from Middle Iranian to Modern Iranian. They briefly mention the case of subject-marking -*eš*, as a limited use in the colloquial register (Jügel and Samvelian 2016: 411–412). They describe this use as restricted to the past tense with the exception of the verb *budan* ‘be’, which they label as an irregular verb, because it lacks a suffix for the 3rd person singular present tense. Jügel and Samvelian (2016) suggest that this use of -*eš* results from a reanalysis process (i.e., reanalysis of pronominal object clitic =*eš* as subject agreement suffix) in order to harmonize/regularize the paradigm by adding an overt form to the only empty slot of the paradigm. They further argue that the prevalence of this -*eš* with intransitive verbs, as opposed to transitive verbs, supports this hypothesis. Their rationale is that when used with intransitive verbs, -*eš* is not a pronominal object, and having no pronominal function, it can only be viewed as a verbal suffix indexing the subject.

Mahootian and Gebhardt’s (2018) study focuses fully on -*eš* (both as object and subject index); however, they are only interested in its status in terms of the clitic/affix distinction and examine it from a strictly synchronic point of view – that is, they do not discuss the development of this marker. The authors present a larger set of examples than in the other studies and, in addition, provide some speaker acceptability judgement data limited to four adults, and gathered in an uncontrolled manner.

Rasekh (2014) proposes a study of Persian pronominal clitics, where he studies their functional evolution. The author discusses -*eš* in particular, describes its use as a subject marker, as “a new trend, absent in careful speech and writing”, and accounts for it in terms of “grammaticalization” of the (object) clitic =*eš* as a means of “repairing the verbal agreement paradigm” (2014: 25). This account is indeed similar to what is also proposed by Jügel and Samvelian (2016), who take an areal and historical view and study Persian within other West-Iranian languages. Rasekh formulates the strongest claims of all, namely that this use is limited to the past tense and to intransitive predicates (2014: 25): he argues that “[since] the subject agreement marker in third singular past tense is zero […], Persian speakers use the clitic, =eš, compensating the absence of subject agreement marker”, and further adds “[t]his =*eš* is used with intransitive predicates, emphasizing its non-argument nature” (Rasekh 2014: 25).

## 3 Research questions

The aim of this paper is to present a usage-based account of subject-marking *-eš* in spoken Persian. Firstly, none of the existing studies have based their claims on data from spoken language usage data. Secondly, up to now, subject-marking *-eš* was either considered solely from an affix-/clitichood point of view, if not ignored, or analysed in terms of grammaticalization. In a nutshell, these studies only considered a few typical examples without any further exploration of other avenues. One of these unexplored avenues is the possibility of viewing the zero/-*eš* alternation as a case of DAI conditioned by a complex interplay of various factors (see, e.g., Just 2022; Just and Čéplö 2022; Walker 2024; Walker et al. 2024 ).

To this end, I will evaluate previous claims against usage data, explore available historical evidence to test the grammaticalization claim, and consider the hypothesis that the zero/-*eš* alternation is part of the indexing system of spoken Persian in contrast to written Persian.

It is worth mentioning that the initial goal of this research was to identify the set of factors responsible for triggering (or favouring) *-eš*. This is clearly a matter of preference and accordingly requires quantitative methods, such as logistic regression or decision trees. Due to the unavailability of richly annotated large-scale corpora (see Section 4.1), this is left for future research – indeed this is a significant methodological challenge, as pointed out in other studies addressing similar research questions (see, e.g., Molochieva et al. 2022).

## 4 Data collection and methodological challenges

### 4.1 Available spoken corpora

There are no richly annotated large-scale corpora of spoken Persian available, and the existing corpora, namely Multi-CAST (Adibifar 2016) and HamBam (Haig and Rasekh-Mahand 2022), do not provide a sufficient basis for a quantitative study. This is because (i) these corpora contain a non-negligible number of transcription and/or glossing inconsistencies with respect to the zero/-*eš* alternation;8To give an example, in Multi-CAST Persian corpus file g2-f-02 (00:04), the first sentence uttered by the speaker contains -*eš* on the first verb *bud* ‘exist’, which is totally absent in the transcription: *Avalaš ke yek mazraei bude …*‘At the beginning, there was a farm …’. The accurate transcription would be *Avaleš ke ye mazraei bud* ***eš*** *…*. Not only do the transcriptions use standard written forms (see underline) instead of what is actually produced by the speaker, it drops extra indexing -*eš*, a feature of colloquial Persian. The HamBam corpus includes two layers of transcriptions. The first layer includes the actual transcription of the utterance, and the second layer a standardized version of it – for instance, the 3rd SG copula *ast*, absent in the spoken version, is systematically put back in the transcription. Unfortunately, the glossing and other annotations are made on this second layer. As for subject marker -*eš*, for example in the file ac_f_social, the speaker frequently produces the construction *in hast*-*eš ke* and both the first and second layers are inconsistent in their transcription: sometime -*eš* is heard but does not appear in either transcriptions, sometime it appears in the first layer but is absent in the second transcription, and sometimes it is indeed present in both layers. This makes it impossible to calculate the rate of zero/-*eš* alternation from this corpus, and for the case of the construction *in hast*-*eš ke* it hides the fact that this form actually alternates with the copular form *in=e ke*, not the standard form *in ast ke*. I’ll come back to this example in Section 5.1. (ii) the zero/-*eš* alternation is not prominent in these corpora, and they cannot provide us with a dataset that is large enough for statistical modelling. It should be noted that this is not solely an issue of size, given that there are genres of spoken discourse, for example, conversations, where zero/-*eš* has a more balanced rate. In other words, there are samples of spoken Persian that could be suitable for our purpose despite their scale. Unfortunately, this is not the case for the samples included in the existing annotated corpora. Accordingly, I decided against using these corpora.

Another option for quantitative/large-scale analyses is the Tehran English-Persian parallel corpus (TEP, Pilevar et al. 2011), a compilation of movie subtitles, available as a part of the OPUS2 parallel corpora collection (Tiedemann 2012). However, this corpus is not an adequate source for a quantitative study of the zero/-*eš* alternation either. This is because: (i) subtitles hardly count as spontaneous language production, (ii) the corpus has no added value in terms of annotation and hence needs to be annotated before the data can be used as input for statistical modelling. Nevertheless, the data could be directly used for comparing production frequencies of different verbs. No matter how the subtitles in this corpus are produced, I assume that they are not likely to present any particular lexical bias interfering with my study of zero/-*eš* alternation. Hence the corpus can be a reasonably reliable indication of usage frequency of -*eš*, and accordingly I have used it for exactly this purpose in this study (see Section 5.3).

### 4.2 Natural spoken data and transcribed spoken data

Spontaneous spoken data is nowadays easy to access on the web or via mobile applications. There are podcasts and similar recordings, to name only a few resources. I hence listened to hours of spontaneous spoken Persian and simply noted down each instance of subject-marking *-eš* – starting with recordings of telephone conversations gathered for a conversation analysis study by Taleghani-Nikazm (1998, 2018) and some (Iranian) reality TV shows.

In the next phase, I focused on spoken data available with Persian transcriptions (provided using the “subtitle” function in YouTube). I used two different sources: TEDx talks delivered and recorded in diverse locations in Iran, and a series of more casual talks with socio-political topics organized by Azad, an independent association of the Sharif University in Tehran. My data includes 22 TEDx talks of about 18 min each, and eight sessions of about two hours each from Azad made available on their YouTube channel from June to October 2023.9
https://www.youtube.com/@azadsocial/about.


This was a matter of convenience, as exploiting data via text output is far less time consuming than via audio output. However, there is a cost for this trade-off: the setting of the language production in these recordings is more or less formal. Nonetheless, interestingly, even in those careful speech settings, where the speaker may use scripted support for their speech, (some) speakers do use subject-marking *-eš*. Given that the use of *-eš* is more common in colloquial speech (than in formal speech), we can assume that those same speakers would have an even higher rate of *-eš* in less formal settings. In other words, observations based on this limited source of data could be safely generalized. Furthermore, as we will see in Section 6, the provided transcription was interesting in its own right.

A complete list with YouTube links is provided in the Appendix, and the transcription texts are provided in the Supplementary Materials. Some of the examples used for illustration in the text come from other sources. In each case the exact source and reference is provided.

## 5 Subject-marking -*eš* in light of usage data

This section presents findings based on a usage study of -*eš* to index 3rd person singular subject on the verb in spoken Persian. First, previous claims to delimit its use are evaluated: restrictions based on tense in Section 5.1 , and on valency in Section 5.2. Next, in Section 5.3, I present quantitative data comparing the rate of *-eš* for a set of verbs. Importantly, lexical restrictions and/or preferences are observed in other cases of DAM cross-linguistically (e.g., Walker and Faghiri 2023; Witzlack-Makarevich and Seržant 2018).

### 5.1 Past versus present stems

As mentioned earlier (Section 2.4), Rasekh (2014) claims that -*eš* as a subject marker is only used in the past tense. This description is, however, not accurate insofar as it ignores the rare but well-established uses of -*eš* in the present tense. There is indeed the obvious exception, *hast* (and its negative counterpart *nist*), the present stem of the verb *budan* ‘exist, be’, which is by all accounts one of the verbs -*eš* is most frequently attested with in spoken contemporary Persian. This is shown in (9), both with a copular function of *nist/hast* (9a) and (9c), and with its existential meaning in (9b) and (9d).

(9)

a.

**Table:** 

*etefāqan*	*ideolojik*	*nist-eš*	*jomhuri=ye*	*eslāmi*
as-a-matter-of-fact	ideologic	neg.cop.prs.3sg-3sg	republic=ez	islamic
‘As a matter of fact, the Islamic Republic is not ideological.’ (Azad recordings)^10^				

10 From Azad recordings https://www.youtube.com/watch?v=8zN4kTKk3Z8 (1:40:33).

b.

**Table:** 

*hič*	*miz=i*	*nist-eš*	*ke*	*post=eš*	*qāem*	
no	table=indf	neg.exist.prs.3sg-3sg	that	behind=3sg	hidden	
*be-š-am*						
sbjv-become.prs-1sg						
‘There is no table (for me) to hide behind.’ (TEDx talk)^11^						

11 From TEDx Tehran talk “Making a Bold Decision” by Reza Pakravan, available on YouTube via https://www.youtube.com/watch?v=P5J179eR7pE (6:25).

c.

**Table:** 

*pedar=e*	*man*	*muzisian*	*hast-eš*
father=ez	1sg	musician	cop.prs.3sg-3sg
‘My father is a musician.’ (TEDx talk)^12^			

12 From TEDx Tehran talk “Make It a Jobby!” by Ramin Sadighi, available on YouTube via https://www.youtube.com/watch?v=atLjDGjIm_A (3:53).

d.

**Table:** 

*agar*	*fazā=ye*	*majāzi*	*hast-eš*	*filtering*	*vojud*	*dār-e*
if	space=ez	virtual	exist.prs.3sg-3sg	filtering	existing	have.prs-3sg
‘If there is the virtual space, there is filtering.’ (Azad recordings)^13^						

13 From Azad recordings https://www.youtube.com/watch?v=kc0fwH8KTXU (6:12).

Interestingly, in my data the most frequent types of constructions with -*eš*, illustrated in (10a), in fact alternate, not with the standard form zero (10c), but with the clitic realization of the copula (10b).14As discussed in Section 4.2, I could not use the two available spoken Persian corpora Multi-CAST and HamBam to calculate frequencies because of their inconsistent transcriptions (see footnote 8). However, I counted the three constructions illustrated in (10) in my TEDx transcription data (a total of more than 53k words) and the results are explicit: 6 cases of *hast-eš ke*, 57 cases of *(h)ast ke* (of which 37 with *ast* and 20 with *hast*), against 110 cases of copular *=e ke*. This is a frequent (sub-)construction in Persian. It appears in different types of cleft constructions as documented in Faghiri and Samvelian’s (2021) corpus-based study. The latter, however, only includes the standard form *in ast ke,* given that their study is based on a standard written corpus.

(10)

a.

**Table:** 

*momken*	*hast-**eš***	*ke*	/	*in*	*hast-**eš***	*ke*	*…*
possible	cop.prs.3sg **-3sg**	that		this	cop.prs.3sg **-3sg**	that

b.

**Table:** 

*momken* ***=e***	*ke*	/	*in=* ***e***	*ke*
possible=cop.prs.3sg	that		this=cop.prs.3sg	that

c.

**Table:** 

*momken*	*ast*	*ke*	/	*in*	*ast*	*ke*
possible	cop.prs.3sg	that		this	cop.prs.3sg	that
‘It is possible that …’				‘(It) is (for) this (reason) that…’

Apart from *hast*, there are still a few other cases that were overlooked by all the above-mentioned studies. As illustrated in (11), -*eš* is attested in the present tense with the verb *āmadan* ‘come’.

(11)

a.

**Table:** 

*dār-e*	*mi-ā-d-eš*	*az*	*safar*
aux.prs-3sg	ipfv-come.prs-3sg-3sg	from	trip
‘(S)he is coming back from (his/her) trip.’ (from a popular song)

b.

**Table:** 

*be*	*omid=e*	*jofte=eš=e*	*ke*	*bāz*	*bi-ā-d-eš*
to	hope=ez	pair=3sg=cop.prs.3sg	that	again	sbjv-come.prs-3sg-3sg
‘(She) is hoping for his other half to come again.’ (from the movie *Khesht-o ayeneh*)

(12)

**Table:** 

*in […]*	*be*	*nazar*	*emkānpazir*	*ne-mi-ā-d-eš*
this	to	mind	possible	neg-ipfv-come.prs-3sg-3sg
‘This … does not seem to be possible.’^15^ (Azad recordings)				

15 From Azad recordings https://www.youtube.com/watch?v=kc0fwH8KTXU (1:09:03).

It is important to mention that this example is by no means exceptional; *mi-ā-d-eš* (or the subjunctive *bi-ā-d-eš*) is frequently heard in spoken Persian, used as a lexical verb ‘come’, as in (11), as well as a light verb, e.g., *be nazar āmadan* ‘seem’, as in (12).16The standard variants of *mi-ā-d* and *bi-ā-d* are respectively *mi-ā-yad* and *bi-ā-yad*. In the spoken variants, [miād] and [biād], the verbal stem /ā/ and person-indexing suffix /ad/ are reduced into a single syllable. It seems reasonable to assume that this simplification has resulted in the speakers’ re-analysis of these forms as *mi-ād-Ø* and *bi-ād-Ø*. Two observations motivate this assumption: firstly, the verb ending for the 3SG in the present tense is the vowel *-e*, the colloquial agreement suffix, and these verbs generally (but not exclusively) have a trisyllabic structure: e.g., *mordan* ‘die’ /mi-mi-re/ (or /be-mi-re/), *residan* ‘arrive’ /mi-re-se/, *raftan* ‘go’ /mi-r-e/, *kardan* ‘do’ /mi-kon-e/, *xordan* ‘eat’ /mi-xor-e/, *zadan* ‘hit’ /mi-zan-e/ etc. Secondly, the consonant /d/ is a frequent ending for the 3SG in the past tense and these verbs generally have a mono- or bi-syllabic structure: e.g., *mordan* ‘die’ /(mi)-mord*-*Ø/, *residan* ‘arrive’ /(mi)-resid*-*Ø/, *raftan* ‘go’ /(mi)-raft*-*Ø/, *kardan* ‘do’ /(mi)-kard*-*Ø/, *xordan* ‘eat’ /(mi)-xord*-*Ø/, *zadan* ‘hit’ /(mi)-zad*-*Ø/ etc. In my collection of spoken Persian data, these are produced by different speakers in different periods and contexts. To give a few examples for illustration: (i) a female character (clearly with a low socio-economic status) living in a poor neighbourhood in Teheran in a movie from the 1960s (*Khesht-o ayeneh* ‘Brick and Mirror’ by Ebrahim Golestan);17
https://en.wikipedia.org/wiki/Brick_and_Mirror, made available online via YouTube (on 25 August 2023) https://www.youtube.com/watch?v=FAzuV12zpaQ (00:08:50). (ii) a middle-aged male university professor as an expert invited speaker in a semi-academic debate setting in 2023.

Another example is given in (13). Here we have the colloquial variant of the formal question *kojā ast* or *kojā-st* ‘where is she/he/it?’, uttered as a single word *kuš* in which the wh-word *kojā* ‘where’ and the present tense 3rd person singular copula *-(a)st* are fused into one syllable. Commonly used in interrogative sentences, *kuš* functions as a simple present stem in colloquial Persian. As shown in (13), *kuš* displays the zero/-*eš* alternation for the 3rd person singular.

(13)

a.

**Table:** 

*bačče-hā*	*kuš-an?*
child-pl	where.cop.prs-3pl
‘Where are the kids?’

b.

**Table:** 

*bačče-hā*	*kuš-in?*
child-pl	where.cop.prs-2pl
‘Kids, where are you?’

c.

**Table:** 

*bačče*	*kuš?*
child	where.cop.prs.3sg
‘Where is the kid?’

d.

**Table:** 

*bačče*	*kuš-eš?*
child	where.cop.prs.3sg-3sg
‘Where is the kid?’^18^	

18 See attested utterance from the movie *Khesht-o ayeneh* (00:24:22): (i)

**Table:** 

*hāšem*	*kuš-eš?*
Hashem	where.cop.prs.2sg-3sg
‘Where is Hashem?’

Finally, in (14) and (15), we have *ināhāš* and *unāhāš,* a pair of verbal expressions from colloquial Persian, similar to *kuš*, which could be viewed as variants of the standard written sentences *injā-st* ‘here she/he/it is’ and *ānjā-st* ‘there she/he/it is’, respectively. They also display the zero/-*eš* alternation for the 3rd person singular.

(14)

a.

**Table:** 

*bačče-hā*	*ināhāš-an!*
child-pl	here.cop.prs-3pl
‘Here are the kids!’

b.

**Table:** 

*bačče*	*ināhāš!*
child	here.cop.prs.3sg
‘Here is the kid!’

c.

**Table:** 

*bačče*	*ināhāš-eš!*
child	here.cop.prs.3sg-3sg
‘Here is the kid!’

(15)

a.

**Table:** 

*bačče-hā*	*unāhāš-an!*
child-pl	there.cop.prs-3pl
‘There are the kids!’

b.

**Table:** 

*bačče*	*unāhāš!*
child	here.cop.prs.3sg
‘There is the kid!’

c.

**Table:** 

*bačče*	*unāhāš-eš!*
child	here.cop.prs.3sg-3sg
‘There is the kid!’

What all these examples share with verbs in the past tense is that they do not carry an audible person index. This implies that the zero/*-eš* alternation is not conditioned/restricted by the tense of the verb, but by its (phonological) form. It is worth emphasizing that these examples, i.e., *hast-eš*, *nist-eš miād-eš*, *ne-miād-eš*, *kuš-eš*, *ināhāš-eš*, *unāhāš-eš*, are by no means infrequent – that is, while they are not the dominant variant, they are not negligible in spoken Persian. While previous studies have not claimed that the tense *per se* is responsible for the use of -*eš* in colloquial Persian, they have nevertheless made inaccurate generalizations. This is problematic, for instance, if we consider the risk for typological studies, because such generalizations could lead to false conclusions when readers are not familiar with the language.

### 5.2 Intransitive (S-marking) versus transitive verbs (A-marking)

According to Rasekh (2014: 25), subject-marking -*eš* is used with intransitive predicates. However, as illustrated by examples in (16), (17) and (18), while the zero/*-eš* alternation mainly occurs with intransitive verbs, it is also possible for non-intransitive verbs. Again, these are by no means exceptional and attested examples for non-intransitive verbs can easily be found.

(16)

a.

**Table:** 

*māhi*_*j*_	*xord-eš*_i/*j_
fish	eat.pst.3sg-3sg
‘(S)he ate fish.’ (Tehran English-Persian parallel corpus)

b.

**Table:** 

*māhi_*j*_	*xord-am=eš*_*j*_
fish	eat.pst-1sg=3sg
(intended)	‘I ate the fish.’

(17)

**Table:** 

*doctor raft-am*	*va doktor*	*goft-eš*	*ke*	…
doctor go.pst-1sg	and doctor	say.pst.3sg-3sg	that	
‘I went to the doctor’s and the doctor said that …’ (HamBam corpus, my glossing)^19^				

19 HamBam corpus file ac_m_depression (00:17), last accessed June 2023. Note that the original glossing does not contain the label A and also has some errors, e.g., go.prs-1sg, instead of go.pst-1sg.

(18)

**Table:** 

*bāqčebun […] *	*did-eš*	*ke yeki*	*az*	*zarf-hā=ye*	*golābi=š*	
gardener	see.pst.3sg-3sg	that one.ptv	of	basket-pl=ez	pear=3sg	
*kam*	šod-e					
less	become.pst-ptcp					
‘The gardener […] saw that one of his pear baskets was missing.’ (MultiCAST, my transcription and glossing)^20^						

20 MultiCAST Persian corpus file g2-f-02 (01:18), last accessed June 2023. Note that *-eš* appears in the transcription (in its standard form *-aš*) glossed as a pronominal clitic and labelled as P.

There is an evident functional explanation for this valency-based preference: with transitive verbs *eš* can be ambiguous between optional subject and object indexing, as in (19). Note that in (16a) -*eš* is not ambiguous and can only be viewed as indexing the subject, since the object, *māhi* ‘fish’, is a (non-specific) bare noun and hence cannot be co-indexed with an anaphoric pronoun. This is shown by the ungrammaticality of (16b), used as a test. The same test excludes this possibility for examples (17) and (18). In these examples, the verb has a clausal object, introduced by the complementizer *ke*.

(19)

**Table:** 

*un*_*i*_ *=o*	*xord-/=eš*_*i/j*_
that=dom	eat.pst.3sg-/=3sg
‘(S)he ate that.’ (Tehran English-Persian parallel corpus)	

It is interesting to note that in the MultiCAST corpus (Adibifar 2016), *-eš* in (18) is originally (mis-)labelled as marking P and thus treated (wrongly) as an object clitic. Indeed, as mentioned previously, the corpus ignores all instances of subject-marking *-eš* on intransitive verbs, while mis-labelling instances of subject-marking *-eš* on transitive verbs. Another example is (20a), cited in Haig (2018: 797), as the only example of (object) clitic doubling in the corpus. Note that the corpus uses a standardized transcription – in other words, utterances are transcribed according to standard Persian and not as actually produced by the speaker.

(20)

a.

**Table:** 

*yek pesar=i […]*	*yeki*	*az*	*ān*	*zanbilhā=rā*	***gozāšt=aš***
one boy=indf	one.ptv	of	those	baskets=dom	put.pst.3sg=3sg(A/P)
*ruye dočarxe=aš*					
onto bike=3sg					
‘[A] boy […] put one of those baskets onto his bike.ʼ (MultiCAST, my glossing)

b.

**Table:** 

??*pesar-hā*	*yeki*	*az*	*ān*	*zanbilhā=rā*	***gozāšt-and=aš***
boy-pl	one.ptv	of	those	baskets= dom	put.pst-3pl=3sg(P)
*ruye dočarxe=ašān*					
onto bike=3pl					
(intended) ‘The boys put one of those baskets onto their bike.’

c.

**Table:** 

??*man*	*yeki*	*az ān*	*zanbilhā=rā*	***gozāšt-am=aš***	*ruye dočarxe=am*
1sg	one.ptv	of those	baskets=dom	put.pst-1sg=3sg(P)	onto bike=1sg
(intended) ‘I put one of those baskets onto my bike.’

Applying the same type of test as in (16), I disagree with this analysis. Changing the subject to the 3rd person plural or to the 1st person singular, I find (20b) and (20c) unacceptable or, at least, clearly less acceptable than (20a). I assume this is due to the fact the object *yeki az ān zanbilhā* does not have a definite reference in the discourse and hence cannot be co-indexed by an anaphoric pronominal clitic.21Note that the presence of the enclitic *=rā* does not imply that the reference is definite, but that it is specific (see, e.g., Faghiri 2016: 18–19). Interestingly, Mahootian and Gebhardt (2018) present some acceptability judgement data that support my observation, against the authors’ own initial predictions. The authors report that “all four speakers that [they] consulted found [example (21)] acceptable, with a preference for interpreting *-eš* as referring to the subject” (2018: 270), while they expected *-eš* to have “a definite/specific interpretation”. It should be noted that Mahootian and Gebhardt (2018) treat subject and object marking *-eš* on a par.

(21)

**Table:** 

*yek*	*gorbe*	*yek*	*muš*	*xord-eš*
a	cat	a	mouse	eat.pst.3sg-3sg
‘A cat ate a mouse.’ (Mahootian and Gebhardt 2018: 270)				

Finally, it is interesting to bear in mind that according to historical studies this use of *-eš* was initially attested with transitive verbs in the past tense (Lazard 1963: 257). The examples provided for illustration are with verbs such as *goftan* ‘say’, *didan* ‘see’, *neveštan* ‘write’, *dādan* ‘give’ and *gereftan* ‘take’. We will return to the historical evidence in Section 7.

### 5.3 Lexical bias

The distribution of zero/-*eš* was compared for different verbs in the Tehran English-Persian parallel corpus (TEP, Pilevar et al. 2011), presented above in Section 4.1, which I accessed via Sketch Engine (Kilgarriff et al. 2014). The list includes four intransitives and four non-intransitives (three transitives and one ditransitive), chosen among the most commonly used (alternating) verbs of the corpus. To obtain token frequencies for each verb, I searched for the 3rd person singular form with and without *-eš*, using the search function in Sketch Engine. For hits with *-eš*, I went through the results and filtered out cases in which *-eš* was not used as a subject marker. The results are presented in Table 2.

**Table 2:** Rate of -*eš* for different verbs – order by their token frequency in TEP corpus.

Verb	Valency	Rate of -*eš*	Number of -*eš*	Total number of tokens
*budan* ‘exist’ pst	intransitive	0.05 %	88	17,557
*šodan* ‘become’	intransitive	0.14 %	10	7,348
*budan* ‘exist’ prs	intransitive	5.64 %	435	7,719
*kardan* ‘do’	transitive	0.04 %	2	4,904
*goftan* ‘say’	ditransitive	2.48 %	75	3,028
*āmadan* ‘come’	intransitive	0.96 %	14	1,464
*raftan* ‘go’	intransitive	0.87 %	11	1,259
*xordan* ‘eat’	transitive	0.04 %	2	466
*āvardan* ‘bring’	transitive	0.02 %	1	386

As expected, we observe a valency-based difference; compare the rate of -*eš* for intransitive verbs like *raftan* ‘go’ or *āmadan* ‘come’ with transitive verbs such as *xordan* ‘eat’ or *āvardan* ‘bring’. In this vein, comparing the rates for *šodan* ‘become’ and *kardan* ‘do’, the two light verbs *par excellence* used to form respectively passive and active predicates, is enlightening: the active LV takes -*eš* about ten times less frequently than the passive LV does.

However, valency does not cover the whole range of variation in the rate of -*eš* observed and there is a level of lexical bias. Firstly, we observe a significantly higher rate of -*eš* with *hast*, the present stem of *budan* ‘exist’, than any other verb. This should not come as a surprise to anybody used to hearing spoken Persian. Secondly, the second most frequent verb to appear with -*eš* is *goftan* ‘say’, a tri-valent predicate, which indexes both A, as in (22a), and P, as in (22b), as well as R (the addressee), as in (22c), in some regional dialects (e.g., Yazd). Attested examples are given in (23), with a given/definite A (23a), as well as an impersonal A (23b).

(22)

a.

**Table:** 

*Ali*_*i*_ *goft-eš*_i/*j_	*ke…*
Ali say.pst.3sg-3sg	that
‘Ali said that …’

b.

**Table:** 

*Ali*_*i*_ *in=o*_*j*_	*goft-eš*_*i/j*_	(cf. *man*_*i*_ *in=o*_*j*_ *goft-am*_*i*_ *=eš*_*j*_)
Ali this=dom	say.pst.3sg-3sg	1sg this=dom say.pst-1sg=3sg
‘Ali said this.’

c.

**Table:** 

*goft-am=eš*_*j*_	*ke…*	(cf. *man*_*i*_	*beh=eš*_*j*_	*goft-am*_*i*_	*ke*)
say.pst-1sg=3sg	that				
‘I told him/her that …’ (Yazdi dialect)

(23)

a.

**Table:** 

*ye āqā=i*	*resid*	*beh=em*	*goft-eš*	*ke*	*če*	*kār*
a man=indf	arrive.pst.3sg	to=1sg	say.pst.3sg-3sg	that	what	work
mi-kon-i?						
ipfv-do.prs-2sg						
‘A man arrived and said to me “what are you doing?”.’^22^ (TEDx talk)						

22 TEDx Tehran talk available on YouTube https://www.youtube.com/watch?v=HUpLLRtoBF4 (8:12).

b.

**Table:** 

*mi-š-e*	*goft-eš*	*ke*	*in*	*vaziat…*
ipfv-become.prs-3sg	say.pst.3sg-3sg	that	this	situation
‘It is possible to say that this situation …’^23^ (Azad recording)				

23 From Azad recordings https://www.youtube.com/watch?v=kc0fwH8KTXU (2:10:20).

Importantly, these high rates cannot be explained based on the verb’s token frequency: compare *goftan* with *kardan* in Table 2. The latter has a much lower rate of -*eš* than *goftan*, despite having a higher token frequency. A possible explanation for the high frequency of -*eš* with *goftan* ‘say’ could be the fact that it is a complement-clause-taking verb. Interestingly, *goftan* ‘say’ and other similar complement-clause-taking verbs (e.g., *didan* ‘see’ and *neveštan* ‘write’) feature in the examples provided in historical studies to show that -*eš* was initially attested with transitive verbs in the past tense (see Section 5.2). This is relevant given that an important part of the use of -*eš* with the copula occurs with complement clauses (see Section 5.1, in particular example (10)). In other words, what the two most frequent (Modern Persian) verbs to appear with -*eš* have in common is that they take complement clauses.24I am thankful to the anonymous reviewer for pointing this connection out to me.


Finally, we observe different preferences among intransitive verbs, which do not correlate with the frequency of these verbs. Considering only the past tense and comparing the four intransitive verbs mentioned above, we have a range from 0.05 % to 1 %, where the highest rate belongs to the less frequent verb of the corpus. In other words, some verbs are more likely to take -*eš* than others, regardless of valency or their frequency of usage.

## 6 Relevance of the spoken modality

A crucial point made in previous studies is the persisting strong resistance of formal/written Persian to the use of subject-marking *-eš*. My data collection confirms this observation: when transcribing spoken data, transcribers frequently correct the speech by dropping extra indexing, according to their personal preference. This is illustrated in (24) and (25), from a TEDx talk.25Available on YouTube via https://www.youtube.com/watch?v=0Yc2tElOv7o (respectively 2:19 and 7:49). The title of the talk is “How Money Is Devaluing Our Wealth?” by Majid Naghedinia. The utterances are presented as transcribed in written Persian, in a, and as produced in b.

(24)

a.

**Table:** 

*در این قرارداد، قرار بود اقتصاد نوین جهان را*
*dar in qarārdad, qarār bud eqtesād novin jahā rā…*

b.

**Table:** 

*tu*	*in*	*qarārdad,*	*qarār*	*bud-eš*	*ke…*
in	this	contract	established	cop.pst.3sg-3sg	that
‘This contract was supposed to …’ (TEDx talk)

(25)

a.

**Table:** 

*این توضیح ساده تورم است*				
*in*	*tozih*	*sāde*	*tavarrom*	*ast*

b.

**Table:** 

*in*	*tozih=e*	*sāde=ye*	*tavarrom*	*hast-eš*
in	explanation=ez	simple=ez	inflation	cop.prs.3sg-3sg
‘This is the simple explanation of inflation.’ (TEDx talk)

This correction goes beyond the use of subject-marking *-eš* and includes other uses of this index, as shown in (26) to (28), from the same source.26YouTube https://www.youtube.com/watch?v=0Yc2tElOv7o (respectively 2:50, 8:15 and 4:01). In the first two examples, the deleted clitic *=eš* corresponds to an object (P argument) index, and in the third example it marks the possessee.

(26)

a.

**Table:** 

*اسکناس‌هایش را … ببرد و به طلا تبدیل کند*		
*eskenās-hā=yaš rā…*	*be-bar-ad*	*va be talā tabdil kon-ad*

b.

**Table:** 

*eskenās=eš=o […]*	*be-bar-e*	*va*	*be*	*talā*	*tabdil* *****=e**š***
banknote=3sg=dom	sbjv-take.prs-3sg	and	to	gold	convert=3sg
kon-e					
do.prs-3sg					
‘[they can] take their banknote […] and convert it to gold.’ (TEDx talk)

(27)

a.

**Table:** 

برای اینکه تورم را بهتر درک کنیم	
*barāye inke tavarrom rā*	*behtar dark kon-im*

b.

**Table:** 

*barāye*	*inke*	*tavarrom=o*	*behtar*	*dark* *****=e**š***	*kon-im*
for	this.that	inflation=dom	better	understand=3sg	do.prs-1pl
‘for our better understanding of inflation.’ (TEDx talk)

(28)

a.

**Table:** 

*آمریکا بیشتر از مقدار موجودی طلا اسکناس چاپ کرده*
*āmrikā bištar az meqdār mojudi talā eskenās čāp karde*

b.

**Table:** 

*āmrikā*	*umad-e:*	*bištar*	*az*	*un*	*meqdār=e*	*mojudi=ye*
America	come.pst-3SG	more	than	that	quantity=ez	stock=ez
*talā* *****=**eš***	*eskenās*	*čāp*	*kard-e:*			
gold=3sg(poss)	banknote	print	do.pst-3SG			
‘The US has printed more banknotes than the quantity of (its) gold stock.’						
(TEDx talk)

A similar correction concerns resumptive pronouns in relative clauses. For instance, *tu=š* ‘in it’, which is frequent in spoken Persian, is regularly replaced with the relative pronoun *ānjā*, as shown in (29) from another TEDx talk.27TEDx University of Tehran talk “Iranian Architecture: A Hidden Treasure” by Taraneh Yalda, available on YouTube https://www.youtube.com/watch?v=r5_qDQxtlfo (2:45).

(29)

a.

**Table:** 

*تورینوکه آنجا درس میخواندم در ایتالیا*						
*torino*	*ke*	*ānjā*	*dars*	*mi-xānd-am*	*dar*	*itāliā…*
Torino	that	where	study	ipfv-read.pst-1sg	in	Italy

b.

**Table:** 

*torino*	*ke*	*tu=š*	*dars*	*mi-xund-am*	*tu itāliā…*
Torino	that	in=3sg	study	ipfv-read.pst-1sg	in Italy
‘… Torino, where I was studying in Italy…’ (TEDx talk)

There is another use of *-eš* that is often corrected in the transcriptions which is not person indexing but, in my view, functions as a tracking device, similar to person indexing, namely for anchoring the utterance in the (previous) speech. In this use, *-eš* is added to different types of adverbials: e.g., *baɁd=eš* ‘afterward (lit. after it)’, *diruz=eš* ‘the day before (lit. its yesterday)’, *fardā=š* ‘the day after (lit. its tomorrow)’, *asl=eš* ‘originally (lit. its origin)’, *haqiqat=eš*/*rāst=eš* ‘to be truthful/truthfully (lit. its truth)’, *vāqeyat=eš* ‘in fact (lit. its fact)’. This is a common and frequent use of *-eš* in colloquial Persian (also see footnote 7).

It is worth noting that these corrections are not limited to the removal of *-eš*. For instance, as illustrated in the pair of examples below from the same TEDx talk,28See https://www.youtube.com/watch?v=r5_qDQxtlfo (respectively 6:38 and 7:10). redundant NP references are also removed in these transcriptions.

(30)

a.

**Table:** 

*…meɁmari=ye*	*mā*	*hanuz…*
[…] architecture=ez	1pl	still
‘… our architecture still …’

b.

**Table:** 

*…meɁmari=ye*	*mā*	*meɁmari=i=e*	*ke*	*hanuz…*
[…] architecture= ez	1pl	architecture=rstr=cop.prs.3sg	that	still
‘… our architecture is an architecture which still …’ (TEDx talk)

(31)

a.

**Table:** 

*…yeki*	*qanāt*	*ast*	*ke…*
[…] one.ptv	qanat	cop.pst.3sg	that
‘… one is qanat which …’

b.

**Table:** 

*…yeki=š*	*qanāt*	*bud-e:*	*ke*	*in*	*qanat-ā…*
[…] one.ptv=3sg	qanat	cop.pst-3sg	that	this	qanat-pl
‘… one of them was qanat that these qanats …’ (TEDx talk)

These are non-trivial observations, given that they highlight the complexity of the indexing system in the spoken language in contrast to the written language. And this is by no means limited to the case of Persian. Indeed, indexing some but not all the subjects on the verb brings to mind the well-known cases of clitic doubling. For example, French clitic doubling (including both subject and object) is a prominent feature of colloquial registers, while being rare or even prohibited in careful/formal registers (see, e.g., Liang et al. 2024  and references therein).

In spontaneous speech, including spoken language as well as online chatting, speakers may rely on these devices to put the main information first, and hence make efficient use of their cognitive/memory resources. Indeed, availability-based incremental models of sentence production postulate that, as long as grammar rules do not intervene, the linear order of constituents reflects the order in which they become available for production: there is a general cognitive preference to put constituents activated at an earlier point in time earlier in the utterance (e.g., Kempen and Harbusch 2003).

In Persian, indexing, as a tracking device, goes hand in hand with word order variations and provides the speakers with significant flexibility in information packaging and, therefore, the possibility to reduce speech planning (cognitive) costs. I have selected four examples from the Azad data29See https://www.youtube.com/watch?v=RAvaPiyeQv4 (respectively 6:57, 17:09, 19:33, 37:20). (32a–d), to showcase this flexibility. These examples illustrate how in spontaneous speech speakers make use of indexing for more efficient information packaging. Utterances are given as produced with the written standard variants added to the right. For instance, in (32a) the noun *budje* ‘budget’ is the topic of the sentence. In the Ezafe construction, *bozorg šod-an=e budje* ‘increasing of the budget’, which is the standard NP construction in Persian (see Samvelian 2007), *budje* appears in the final position. However, there is the possibility of placing it first, using an alternative order with the help of indexing: *budje*_*i*_ *bozorg šod-an=eš*_*i*_.

(32)

a.

**Table:** 

*budje*_*i*_	*bozorg šod-an=eš*_*i*_	*…*	vs.	*bozorg*	*šod-an=e*
budget	large idem-inf=3sg			large	become-inf=ez
*budje …*					
budget					
‘The budget, its increasing … /The increasing of the budget…’

b.

**Table:** 

*man*_*i*_ *tarjih=am*_*i*_	*in=e*	vs.	*tarjih=e*	*man*
1sg preference=1sg	this=cop.prs.3sg		preference=ez	1sg
‘Me, what I prefer is this. / My preference is this.’

c.

**Table:** 

*vaqti entexābāt*_*i*_	*natij=aš*_*i*_	*elām*	*šod*	*vs. natije=ye*
when elections	result=3sg	announce	become.pst.3sg	result=3sg
*entexābāt*				
elections				
‘When the elections, its results / the results of the elections were announced.’

d.

**Table:** 

*benzin*_*i*_	*ke*	*xod=eš*_*i*_	*qeymat=eš*_*i*_	*ne-mi-r-e*	*bālā*	vs. *qeymat=e*
petrol	that	self=3sg	price=3sg	neg-ipfv-go.prs-3sg	up	price=ez
*benzin*						
petrol						
‘The price of petrol doesn’t go up by itself.’ (Azad recordings)

Constructions such as those illustrated in (32) are frequent in spontaneous speech, even in these relatively careful speech contexts. Otherwise, in offline language production, where planning costs are less of an issue, language users voluntarily drop the indexes and use canonical word orders. This contrast is again illustrated by the corrected transcriptions, as illustrated in (33) from the same talk.30YouTube https://www.youtube.com/watch?v=r5_qDQxtlfo (9:02).

(33)

a.

**Table:** 

*به آن می‌گویند آرت دکو، سبکی که در واقع بعد از رضاشاه درست شده*						
*be*	*ān*	*mi-guy-and*	*ār*	*deko*	*sabk=i*	*ke*
to	that	ipfv-say.prs-3pl	Art	Deco	style=rstr	that
*dar-vāqeɁ*	*baɁd*	*az*	*rezā šāh*	*dorost*	*šod-e:*	
in-fact	after	from	Reza Shah	made	become.pst-3sg	
‘They call it Art Deco, a style which was in fact introduced after Reza Shah.’

b.

**Table:** 

*beh=š*	*mi-g-an*	*ar*	*deko*	*esm=e*	*sabk=eš=e*		
to=3sg	ipfv-say.prs-3pl	Art	Deco	name=ez	style=3sg=cop.prs.3sg		
*va*	*dar*	*dore=ye*	*dar-vāqeɁ*	*rezā*	*šāh*	*dorost*	*šod-e:*
and	in	period=ez	in-fact	Reza	Shah	made	become.pst-3sg
*be*	*baɁd*						
to	after						
‘They call it Art Deco, it is the name of its style and it was in fact introduced in Reza Shah period afterwards.’ (TEDx talk)

In (33), beyond the usual changes in lexical and morphological levels, we observe different types of corrections, including word order and index drop. In the utterance as produced in (33b), to explain *Art Deco* the speaker uses a copular clause with a possessive construction (*esm=e sabk=eš=e* ‘it is the name of its style’), followed by another simple clause (*va dar dore=ye dar-vāqeɁ rezā šāh dorost šode be baɁd* ‘and is made in fact in the period after Reza Shah’). In the transcription (33a), these clauses are replaced by a relative clause: *sabk=i ke baɁd az rezā šāh dorost šode* ‘a style which is made after Reza Shah’. In addition, in the transcription the speaker’s hesitation, shown in the late utterance of *be baɁd* ‘afterwards’, is omitted and rendered with a simplified order *baɁd az rezā šāh* ‘after Reza Shah’.

## 7 Optional subject indexing in spoken Persian

In Section 5 and 6, I outlined empirical shortcomings of previous studies and further provided empirical evidence to highlight the importance of the spoken modality in the analysis of *-eš*. Building on insights from language processing, I showed that the difference between written and spoken Persian is indeed relevant for the issue at stake. This evidence suggests that like many other languages with a standard written form and literary tradition, indexing in spoken Persian is more complex, including variations that are absent from standard written Persian. In the present section, I will further evaluate the alternative view that subject-marking -*eš* is a means of harmonizing the verbal agreement paradigm.

I focus on Rasekh (2014), which, to my knowledge, proposes the most explicit account of subject-marking -*eš* in Modern Persian in terms of grammaticalization, claiming that “the grammaticalization of [the] clitic has repaired this ‘defect’ of the paradigm” (2014: 25) – where “defect” refers to the fact that the paradigm has no phonological form for 3SG. In particular, I will show that his account faces at least two serious challenges. First, it does not cover the alternation. In other words, it is not the case that the verbal agreement paradigm is indeed “repaired”. If -*eš* is to be viewed as an agreement suffix on a par with other agreement suffixes in the same paradigm, then the present state should be considered as transitional and we should expect the use of -*eš* to be generalized (fully overtaking zero) at some future stage. I, however, do not think that the existing evidence supports this prediction, as I will show that it actually suggests the opposite. In any case, Rasekh (2014) does not provide any historical data or evidence to support this claim. This is even more problematic given the assumption of this “repair” being a *new* development is also taken for granted. And this is the second issue I will discuss below.

As noted in existing descriptive grammars (e.g., Vahidian Kamyar 1964), it is rather the index drop which is a more recent trend and a particularity of formal/written Persian, whereas -*eš* has been a feature of Modern Persian since its early days.31“Old Dari” (my translation of *zabān-e dari-e qadim*), in Vahidi Kamyar’s own words. Given that Dari is another term to refer to Modern Persian (aka New Persian), I assume that the author is referring to Early Modern Persian. Furthermore, it is prominent in some regional dialects, for instance those spoken in Khorasan, including the Mashhadi dialect (Vahidian Kamyar 1964: 36). Importantly, according to Lazard (1963: 257–258), this use of -*eš*, which he qualifies as *pléonastique*, is attested already in Early New Persian (8th to 12th centuries), leading Paul (2019: 592) to suggest that “[it] may always have existed, throughout the history of Persian as a possible albeit (until today) non-preferred sub-standard construction”.

The extended usage of 3rd person singular and plural suffixes for representing the subject (both with intransitive and transitive, in the past as well as in the present tense) is conserved well into the modern period, where this use is frequent in the colloquial Persian of Tehran. (Lazard 1963: 257 [my translation])32“L’emploi étendu des suffixes de la 3e personne singulier et pluriel pour représenter le sujet (tant des verbes intransitifs que des verbes transitifs et au présent comme au passé) s’est conservé jusqu’à l’époque moderne, où il est usuel dans le persan familier de Téhéran”. Indeed, Lazard (1963: 257–258) provides a few examples with 3rd person plural added to a verb in the present tense already carrying the 3rd person plural (subject) suffix, e.g., *bi-dān-and-išān* (sbjv-know.prs-3pl-3pl) ‘for them to know’ (‘qu’ils sachent’ in French).

Moreover, I follow Haig’s (2018) reasoning and call into question an argumentation that would take historical evidence from *written corpora* as diachronic justification for considering a feature of *spoken language* as a new development. Haig refutes object clitic doubling in Persian and argues against van Gelderen (2011), who views clitic object pronouns in Persian as developing into agreement markers, by questioning the assumption that they are new (an “innovation” in van Gelderen’s term), because “[w]e simply lack direct attestation of colloquial spoken Persian prior to the twentieth century, so it is perfectly possible that the kind of sporadic clitic doubling found in modern spoken Persian has been available in colloquial registers for centuries, but never found its way into writing, just as the modern clitic doubling is seldom found in the contemporary written language” (2018: 797).

In addition, putting aside the function of -*eš* in the Modern Persian agreement system, it is clear that given the diachronic evidence its use cannot strictly speaking be considered “new”, as it appears to be a relic of earlier stages of subject indexing or “des vestiges de cette construction” according to Lazard (1963: 257). Earlier stages of (West)-Iranian languages, namely Middle Iranian languages, including Middle Persian (3rd to 8th century), deployed a quasi-identical paradigm of (second position) pronominal clitics for indexing the P argument in the present tense (34), and the A argument in the past tense (35) (see, e.g., Haig 2018: 798–799).33These paradigms are not fully identical but considered cognates at the very least (e.g., Haig 2020: 93).

(34)

**Table:** 

*[…] u* ***=š***	*hamēw*	*bōžēnd*	*[…]*
and**=3sg:P**	always	save.pres.3pl	
‘[The Gods] always saved him.’ (Originally from Haig 2008, reproduced here from Haig 2018:795)

(35)

a.

**Table:** 

*u* ***=š***	*ardawān*	*ōzad*	*…*
and**=3sg:A**	Ardawān	kill.pst.3sg	
‘And he_i_ killed Ardawān …’ (Originally from Jügel 2015, reproduced here from Haig 2018: 799)

b.

**Table:** 

*u* *****=**š***	***guft***	*dādār*	*ohrmazd*	*kū…*
and= **pc.3sg**	said. **pst**	creator	Ohrmazd	that
**‘and he said, the creator Ohrmazd, that …’**

c.

**Table:** 

***guft=iš***	*ohrmazd*	*ō*	*spitāmān*	*zardušt*	*kū…*
said. **pst=pc.3sg**	Ohrmazd	to	Spitāmān	Zardušt	that
‘and he said, Ohrmazd, to Spitāmān Zardušt that …’ (Originally from Jügel 2015, reproduced here from Jügel and Samvelian 2020: 140)^34^					

34 The glosses are reproduced from the references. Note that in examples from Jügel and Samvelian (2020) “pc” stands for “clitic pronoun”.

Haig (2018) further shows that the same pronominal paradigm has fully developed into subject agreement in Central Kurdish: “In Central Kurdish, the system is still recognizably that of Middle Iranian, but with one very crucial difference: the pronominal clitics that index an A in the past tenses have become fully obligatory” (2018: 799). The following examples are originally provided to illustrate the case of object clitics in the Mukri dialect of Central Kurdish, but they also provide an illustration for A indexing in the past tense, which also shows the similarity with Middle Iranian nicely (compare (35c) to (36a)).

(36)

a.

**Table:** 

***kut=ī***	*“segbāb*	*bo*	*de=m=guž-ī?”*
say.pst=**3sg.A**	dog.son	why	indic=1sg.P=kill.prs-2sg
‘He said: “Son of a dog, why are you killing me?”.’

b.

**Table:** 

***kut=im***	*“bāb=im*	*nā=t=guž-im”*
say.pst=**1sg.A**	brother=poss.1sg	neg=2sg.P=kill.prs-1sg
‘I said: “O brother, I am not killing you”.’ (Originally from Öpengin 2016, reproduced here from Haig 2018: 796)

Hence, in terms of the development of the indexing system after the stabilization of the alignment change from ergative (in the past tense) to accusative in Modern Persian (see, e.g., Jügel and Samvelian 2020: 137–138), it seems more feasible to assume that zero marking for the 3rd person singular resulted from simplification of the system. On the one hand, in a six-variable paradigm, the degree of freedom is five, and one value could be dropped. Indeed, this observation should be sufficient by itself to exclude the repairing hypothesis since the system is already (structurally) perfect as it is. However, it does not exclude the “levelling” or “harmonizing” hypothesis (Jügel and Samvelian 2016: 411).

On the other hand, it is a well-established typological observation that zero marking for the 3rd person is common cross-linguistically. It seems reasonable, given the historical evidence, to assume that whereas the zero/*-eš* alternation continued in colloquial (and/or vernacular) registers, the simplified indexing paradigm, with fixed zero indexing of 3SG, was established as the unified norm promoted as standard Persian with the diffusion of formal written language. This may well be due to an inclination towards a fully transparent (and economical) indexing device – in other words a parsimonious system. The same can be observed with respect to word order. As noted in historical studies (e.g., Khānlari 1994: 18–21 and 266–267), starting from a free word order language, standard Persian has become much more rigid where colloquial Persian shows a greater degree of variation (see, e.g., Faghiri 2016: 30–35).

In addition, there is no evidence to suggest that the zero/*-eš* alternation would at some point develop into a six-variable paradigm with *-eš* fully replacing zero. Recall that *-eš* is a relic of the indexing system in Middle Persian, after the stabilization of the alignment change from ergative to accusative in Modern Persian. This alignment change resulted in (i) a unified behaviour for subject marking in transitive and intransitive predicates, and (ii) a unified behaviour for object marking in past and present tenses. It is reasonable to assume that these unified behaviours are structurally optimal. Now let us consider the two options for 3rd person singular subject marking, i.e., fully non-alternating *-eš* and fully non-alternating zero, in this respect. Firstly, while zero presents no ambiguity whatsoever, *-eš* is only unambiguous with intransitive predicates. Secondly, in a non-alternating zero system, there is only one clitic *=eš*. Hence object indexing operates in exactly the same way for present and past tenses (see Table 1). However, this is no longer the case in a non-alternating *-eš* system: with present stems it is possible to index both a 3rd person singular subject and object on the verb filling two different (but fixed) slots: *mi-xor-ad*_*A*_ *=eš*_*P*_ ‘s/he eats it’, but with past stems only one slot is available for this combination, mi-*xord-eš*_*A/P*_ ‘s/he ate (it)’. This means that a system with a non-alternating *-eš* is less systematic/harmonious and displays structural ambiguities. In other words, the prediction that *-eš* would ultimately be fully generalized (in order to achieve a more harmonious system) is not empirically well-grounded, simply because a system with a non-alternating *-eš* has fewer structural advantages over a system with a non-alternating zero.

At this point, it is worth mentioning that there is a case of reanalysis (and hence grammaticalization) in the verbal paradigm in colloquial Persian, which, as I will show below, is completely well-founded empirically: subject agreement in the present perfect. The latter is built with the past participle and the copula, as in (37a) and (38a). The past particle is formed with the suffix *-e* added to the past stem, e.g., *rafte* ‘gone’, *gofte* ‘said’, *dide* ‘seen’. In spoken Persian, the copular clitics are amalgamated with the stem (Paul 2019: 607) and result in one final syllable (37b and 38b). Consequently, in spoken Persian, except for the 3rd person singular, the difference between the present perfect (37b), and simple past tenses (37c), is reduced to the length of the final syllable and/or the position of the stress, which is the last syllable for the present perfect. Stress follows the general rule for the simple past and falls on the final syllable of the verbal stem, and the verbal suffix remains unstressed (see Paul 2019: 585).

(37)

a.

**Table:** 

*di′d-e=and*
see.pst-ptcp=cop.prs.3pl

b.

**Table:** 

*di′d-a:n*
‘They have seen.’

c.

**Table:** 

*′did-an*
see.pst-3pl
‘They saw.’

(38)

a.

**Table:** 

*di′d-e=ast*
see.pst-ptcp=cop.prs.3sg

b.

**Table:** 

*di′d-e:*
‘(S)he has seen.’

c.

**Table:** 

*′did-Ø / ′did-eš*
see.pst.3sg /see.pst-3sg
‘(S)he saw.’

For the 3rd person singular, in the spoken language (38b), the copula is fully dropped, and the stress falls on the last vowel, the suffix *-e*.35The grammaticalization or levelling hypotheses do not warrant the appearance of an empty slot in the paradigm, as in that view it would make the paradigm defective or unbalanced. The difference between the simple past and the present perfect is hence segmental as well as stress-based. What is further interesting for the issue at stake here is the fact that, even though in the present perfect tense the 3rd person singular bears no person/number marker in colloquial Persian, speakers never use *-eš* (or any other clitic or affixes for that matter). This is hence a case of grammaticalization in colloquial Persian: the past participle suffix *-e* is reanalysed as a person/number marker for the 3rd person singular in the present perfect. This undermines the grammaticalization hypothesis in the case of *-eš*. On the contrary, considering the verbal paradigm more globally, it seems reasonable to postulate that in colloquial Persian *-eš* (partly) materializes the distinction between the simple past and the present perfect tenses for the 3rd person singular.

Finally, one last piece of evidence to exclude the grammaticalization of *-eš* as an agreement marker is the fact that it does not behave like other members of the same paradigm. Assuming, for the sake of the argument, that *-eš* is grammaticalized as a subject agreement marker, it would belong to the same paradigm as the 1st person singular marker *-am* (39) or the 3rd person plural marker *-an* (40). However, as illustrated by the ungrammaticality of (41b), *-eš* does not share all the distributional features of the members of this paradigm. While there may be valid arguments to explain the different combinatory behaviour of *eš*, the argument against the grammaticalization hypothesis remains: levelling or repairing implies that the expected endpoint (repaired/levelled) result would be a regular homogeneous paradigm, but this is clearly not the case.

(39)

a.

**Table:** 

*to=ro*	*did-am*
2sg=dom	see.pst-1sg

b.

**Table:** 

*did-am=et*
see.pst-1sg=2sg
‘I saw you.’

(40)

a.

**Table:** 

*to=ro*	*did-an*
2sg=dom	see.pst-3pl

b.

**Table:** 

*did-an=et*
see.pst-3pl=2sg
‘They saw you.’

(41)

a.

**Table:** 

*to=ro*	*did-eš*
2sg=dom	see.pst-3sg

b.

**Table:** 

*did-eš=et
see.pst-3sg=2sg
‘(S)he saw you.’

In sum, there is no empirical evidence to support an account of *-eš* in terms of grammaticalization and/or levelling of the verbal agreement paradigm. On the contrary, all the evidence leads to the conclusion that the zero/*-eš* alternation is a feature of the indexing system in spoken Persian, while absent from written Persian. In other words, optionally indexing subjects on the verb using *-eš* is a case of DAI and is limited to the colloquial register and/or spontaneous speech.

Written Persian differs from spoken Persian in various syntactic aspects; word order is a well-known feature; another one, highlighted in this study, is argument indexing, in particular, subject indexing. The case of object clitic doubling (see, e.g., (19) in Section 5.2, (26) and (27) in Section 6) is also fairly well-known in the existing literature (see, e.g., Haig 2018: 797; 2020: 100–101). Indeed, in Section 6 we saw that object clitic doubling also exemplifies a case of ‘extra’ indexing in spoken Persian – as opposed to the written language. Interestingly, discrepancies between written (and/or prescriptive) and spoken language in terms of differential indexing have been noticed before (Just and Čéplö 2022). Hence both cases may be viewed as forms of DAI, and the indexing system in Persian could be classified as “split-fluid” in Witzlack-Makarevich and Seržant’s (2018: 28) terms.

## 8 Conclusions

In this study, I presented a comprehensive overview of subject-marking *-eš* in colloquial Persian. While using *-eš* to index the 3rd person singular subject is prohibited by the prescriptive norm, and hence is absent from standard written Persian, it is considered a non-preferred sub-standard construction or a pleonastic use in colloquial Persian by descriptive grammars. Previous research in theoretical linguistics, however, has mostly ignored or neglected this use. The few studies that exist either focus on the clitic/affix properties of the marker or account for it superficially in terms of grammaticalization, making empirically unwarranted claims. My study builds on a sizeable sample of natural data from spoken Persian as well as evidence from historical studies.

I reviewed previous claims and highlighted their shortcomings, and argued against grammaticalization accounts by showing that their hypotheses are not supported by any historical or synchronic evidence. The historical evidence allows us to identify the 3SG subject index -*eš* as a relic of an earlier stage of Persian’s agreement system, and the zero/-*eš* alternation as a feature of Modern Persian since its early days. Importantly, my study highlights the importance of the spoken modality in the analysis of *-eš*, a fact taken for granted previously. To this end, I presented data from spoken Persian transcriptions, made available as subtitles in video recordings in two different contexts. In a nutshell, I compared spoken utterances with their written transcriptions and identified patterns of difference in indexing between the two modalities. I argued that it is possible to explain these patterns on the basis of cognitive principles established by studies in language planning and processing. Spontaneous language production in the spoken modality differs in effect from offline language production in the written modality in terms of utterance planning. Importantly, the spoken modality favours constructions that are efficient with regard to information packaging, and are hence less costly for working memory. The written modality, on the contrary, favours parsimonious constructions regardless of their complexity or cognitive cost. Accordingly, I claimed that extra indexing, namely the zero/*-eš* alternation, should be considered a feature of the indexing system in spoken Persian, as opposed to written Persian. In this vein, the (redundant) use of *-eš* to index the subject, similar to object clitic doubling, could be accounted for in terms of differential indexing. Future research, using a robust quantitative methodology, should shed further light on the triggers of these alternations in spoken Persian.

## Supplementary Material

Supplementary Material

## Glossing abbreviations

1: first person
2: second person
3: third person
a: agent-like argument of a transitive verb
aux: auxiliary
cop: copula
cl: clitic
dom: differential object marker
ez: ezafe construction
ipfv: imperfective
indf: indefinite
inf: infinitive
neg: negation
p: patient-like argument of a transitive verb
pl: plural
poss: possessive
prs: present
pst: past
ptcp: participle
ptv: partitive
rstr: restrictive
sg: singular
sbjv: subjunctive

## Appendix

YouTube video links.

### TEDx talks

Shahrzad Esfarjani https://www.youtube.com/watch?v=jc_hybX94T4.

Sarvenaz Heraner https://www.youtube.com/watch?v=Og7jXXMyvWg.

Ehsan Jahandarpour https://www.youtube.com/watch?v=DK2RqXKAJhM.

Gelareh Kiazand https://www.youtube.com/watch?v=i6pHcmTWgAs.

Nasrin Moazami https://www.youtube.com/watch?v=zX7Dyqidcj8.

Majid Naghedinia https://www.youtube.com/watch?v=0Yc2tElOv7o.

Mashallah Naghilou https://www.youtube.com/watch?v=HUpLLRtoBF4.

Saba Nassiri https://www.youtube.com/watch?v=iuUov_fYFgE.

Reza Pakravan https://www.youtube.com/watch?v=P5J179eR7pE.

Paniez Paykari https://www.youtube.com/watch?v=gq0sshXiNVs.

Pooye Pezeshkirad https://www.youtube.com/watch?v=xr1QSneY0tg.

Nima Rezaei https://www.youtube.com/watch?v=TtnEZUN8-bQ.

Ramin Sadigh https://www.youtube.com/watch?v=atLjDGjIm_A.

Reza Sayah https://www.youtube.com/watch?v=16CEPz9rQ2E.

Alireza Shafaghinejad https://www.youtube.com/watch?v=-H6xqeWlgq4.

Kiana Shafiei https://www.youtube.com/watch?v=prhWJxpO6yg.

Shahrzad Shokouhivand https://www.youtube.com/watch?v=uBegzy_iN1Y.

Farid Shokrieh https://www.youtube.com/watch?v=SNA9JGZf8T8.

Taraneh Yalda https://www.youtube.com/watch?v=r5_qDQxtlfo.

Shabnam Yazdani https://www.youtube.com/watch?v=cd8f2KIx1e4.

Rabe’eh Zand https://www.youtube.com/watch?v=wp4hSmOw2ZA.

### Azad dialogues

Mohammad Māldjou https://www.youtube.com/watch?v=kc0fwH8KTXU.

Masoud Nili https://www.youtube.com/watch?v=RAvaPiyeQv4.

Abbās Abdi and Sinā Kalhor.

Short clip 1 https://www.youtube.com/watch?v=UJDQ71QFgnQ.

Short clip 2 https://www.youtube.com/watch?v=dr6lUxuJJxU

Bijan Abdolkarmi and Madjid Tavakkoli https://www.youtube.com/watch?v=z-Y-G0dxCZk.

Mostafā Malekiān https://www.youtube.com/watch?v=t6QEXvLlJWI.

Morād Saqafi https://www.youtube.com/watch?v=-vVCKyuocwo.

Mehdi Khaladji and Ahmad Zeidābadi https://www.youtube.com/watch?v=8zN4kTKk3Z8.

Abbās Amānat https://www.youtube.com/watch?v=xs4tPep-TWE.

## References

[j_ling-2023-0235_ref_001] Adibifar Shirin, Haig Geoffrey, Schnell Stefan (2016). Multi-CAST Persian. *Multi- CAST: Multilingual corpus of annotated spoken texts*.

[j_ling-2023-0235_ref_003] Degen Judith, Hawkins D. Robert, Graf Caroline, Kreiss Elisa, Goodman Noah D. (2020). When redundancy is useful: A Bayesian approach to “overinformative” referring expressions. *Psychological Review*.

[j_ling-2023-0235_ref_011] de Hoop Helen, de Swart Peter (2008). *Differential subject marking*.

[j_ling-2023-0235_ref_004] Faghiri Pegah (2016). *La variation de l’ordre des constituants dans le domaine préverbal en persan: Approche empirique*.

[j_ling-2023-0235_ref_005] Faghiri Pegah, Samvelian Pollet (2021). A corpus-based description of cleft constructions in Persian. *Faits de Langues*.

[j_ling-2023-0235_ref_006] Haig Geoffrey, Coon Jessica, Massam Diane, Travis Lisa (2017). Deconstructing Iranian ergativity. *The Oxford handbook of ergativity*.

[j_ling-2023-0235_ref_007] Haig Geoffrey (2018). The grammaticalization of object pronouns: Why differential object indexing is an attractor state. *Linguistics*.

[j_ling-2023-0235_ref_008] Haig Geoffrey, Larson Richard K., Moradi Sedigheh, Samiian Vida (2020). The pronoun-to-agreement cycle in Iranian: Subjects do, objects don’t. *Advances in Iranian linguistics*.

[j_ling-2023-0235_ref_009] Haig Geoffrey, Rasekh-Mahand Mohammad (2022). *HamBam: The Hamedan-Bamberg Corpus of contemporary spoken Persian*.

[j_ling-2023-0235_ref_010] Haspelmath Martin, Bakker Dik, Haspelmath Martin (2013). Argument indexing: A conceptual framework for the syntactic status of bound person forms. *Languages across boundaries: Studies in memory of Anna Siewierska*.

[j_ling-2023-0235_ref_012] Jügel Thomas (2015). *Die Entwicklung der Ergativkonstruktion im Alt- und Mitteliranischen – eine korpusbasierte Untersuchung zu Kasus, Kongruenz und Satzbau*.

[j_ling-2023-0235_ref_013] Jügel Thomas, Samvelian Pollet (2016). Les pronoms enclitiques dans les langues ouest-iraniennes. *Bulletin de la Societe de Linguistique de Paris*.

[j_ling-2023-0235_ref_014] Jügel Thomas, Samvelian Pollet, Larson Richard K., Moradi Sedigheh, Samiian Vida (2020). Topic agreement, experiencer constructions, and the weight of clitics. *Advances in Iranian linguistics*.

[j_ling-2023-0235_ref_015] Just Erika (2022). *A functional approach to differential indexing: Combining perspectives from typology and corpus linguistics*.

[j_ling-2023-0235_ref_016] Just Erika, Čéplö Slavomír, Turek Przemyslaw, Nintemann Julia (2022). Differential object indexing in Maltese: A corpus based pilot study. *Maltese: Contemporary changes and historical innovations*.

[j_ling-2023-0235_ref_017] Kempen Gerard, Harbusch Karin, Hartl Holden, Tappe Heike (2003). Word order scrambling as a consequence of incremental sentence production. *Mediating between concepts and grammar*.

[j_ling-2023-0235_ref_018] Khānlari Parviz Nātel (1994). *Dastur-e tārixi-ye zabān-e fārsi [A historical grammar of Persian]*.

[j_ling-2023-0235_ref_019] Kilgarriff Adam, Baisa Vít, Bušta Jan, Jakubíček Miloš, Vojtěch Kovář, Michelfeit Jan, Rychlý Pavel, Suchomel Vít (2014). The Sketch engine: Ten years on. *Lexicography*.

[j_ling-2023-0235_ref_020] Lazard Gilbert (1957). *Grammaire du persan contemporain*.

[j_ling-2023-0235_ref_021] Lazard Gilbert (1963). *La langue des plus anciens monuments de la prose persane*.

[j_ling-2023-0235_ref_022] Lazard Gilbert (1998). *Actancy*.

[j_ling-2023-0235_ref_043] Liang Yiming, Donati Caterina, Burnett Heather (2024). French subject doubling: A third path.. *Isogloss. Open Journal of Romance Linguistics*.

[j_ling-2023-0235_ref_023] Mahootian Shahrzad, Gebhardt Lewis, Korangy Alireza, Miller Corey (2018). Revisiting the status of -eš in Persian. *Trends in Iranian and Persian linguistics*.

[j_ling-2023-0235_ref_024] Miller Jim, Fernandez-Vest Jocelyne, Bernini Giuliano, Schwartz Marcia L. (2006). Spoken and written language. *Pragmatic organisation of discourse in languages in Europe*.

[j_ling-2023-0235_ref_025] Molochieva Zarina, Faghiri Pegah, van Lier Eva (2022). Biabsolutive constructions in Chechen. *Folia Linguistica*.

[j_ling-2023-0235_ref_026] Öpengin Ergin (2016). *The Mukri variety of Central Kurdish: Grammar, texts and lexicon*.

[j_ling-2023-0235_ref_027] Paul Ludwig, Haig Geoffrey, Khan Geoffrey (2019). Persian. *The languages and linguistics of Western Asia: An areal perspective*.

[j_ling-2023-0235_ref_028] Pilevar Mohammad Taher, Faili Heshaam, Abdol Hamid Pilevar (2011). TEP: Tehran English-Persian parallel corpus. *CICLing*.

[j_ling-2023-0235_ref_029] Rasekh Mohammad (2014). Persian clitics: Doubling and agreement. *Journal of Modern Languages*.

[j_ling-2023-0235_ref_030] Samvelian Pollet (2007). A (phrasal) affix analysis of the Persian ezafe. *Journal of Linguistics*.

[j_ling-2023-0235_ref_031] Seržant Ilja A., Witzlack-Makarevich Alena (2018). *Diachrony of differential argument marking* (Studies in diversity linguistics 19).

[j_ling-2023-0235_ref_032] Taleghani-Nikazm Carmen (1998). Politeness in Persian interaction: The preference format of offers in Persian. *Cross-roads of Language, Interaction, and Culture*.

[j_ling-2023-0235_ref_033] Taleghani-Nikazm Carmen (2018). Invitations in Farsi: An analysis of their turn formats and sequential organizations. *Journal of Pragmatics*.

[j_ling-2023-0235_ref_034] Tiedemann Jörg (2012). Parallel data, tools and interfaces in OPUS. *Proceedings of the 8th international conference on language resources and evaluation (LREC’2012)*.

[j_ling-2023-0235_ref_035] Vahidian Kamyar Taghi (1964). *Dastur-e zabān-e āmiāne-ye fārsi [A grammar of colloquial Persian]*.

[j_ling-2023-0235_ref_036] Vahidian Kamyar Taghi (2005). *Dastur-e zabān-e goftāri-ye fārsi [A grammar of spoken Persian]*.

[j_ling-2023-0235_ref_037] van Gelderen Elly (2011). *The linguistic cycle: Language change and the language faculty*.

[j_ling-2023-0235_ref_038] Walker Katherine (2024). *Conditional indexing*.

[j_ling-2023-0235_ref_039] Walker Katherine, Pegah Faghiri, Eva van Lier (2024). Argument indexing in Kamang. *Studies about*. *Languages*.

[j_ling-2023-0235_ref_040] Walker Katherine, Faghiri Pegah (2023). Introduction to lexical constraints in grammar: Minority verb classes and restricted alternations. *Open Linguistics*.

[j_ling-2023-0235_ref_042] Witzlack-Makarevich Alena, Seržant Ilja A., Seržant Ilja A., Witzlack-Makarevich Alena (2018). Differential argument marking: Patterns of variation. *Diachrony of differential argument marking*.

