# Supplementary material for: Optional subject indexing in spontaneous speech in Modern Persian
Source: Linguistics. 2026 Feb 20;64(3):603–38. doi: 10.1515/ling-2023-0235 (PMC13267781; doi:10.1515/ling-2023-0235)
Supplement: Supplementary file 1 — Supplementary Material [file j_ling-2023_0235_suppl_001.docx]

Supplementary Material

Full texts of transcribed data for each video listed can be find at the following Open Science Framework link: <https://osf.io/k8qa3/?view_only=e4a7835eb16441868e337ad3cd618729> <https://osf.io/k8qa3/?view_only=9740bf1ac36a41cea08d02e2c9533b8d>
